# Health-related quality of life in the phase III GALLIUM study of obinutuzumab- or rituximab-based chemotherapy in patients with previously untreated advanced follicular lymphoma

**DOI:** 10.1007/s00277-020-04021-6

**Published:** 2020-04-20

**Authors:** Andrew Davies, Peter Trask, Judit Demeter, Axel Florschütz, Mathias Hänel, Tomohiro Kinoshita, Ruth Pettengell, Hang Quach, Stephen Robinson, Shalal Sadullah, Juan-Manuel Sancho, Miklos Udvardy, Mathias Witzens-Harig, Andrea Knapp, Wenxin Liu

**Affiliations:** 1 Cancer Research UK Centre, Centre for Cancer Immunology, Faculty of Medicine, University of Southampton, Southampton General Hospital, Southampton, SO16 6YD UK; 2 Genentech Inc., South San Francisco, CA USA; 3 Semmelweis University, Budapest, Hungary; 4 Städtisches Klinikum Dessau, Dessau-Roßlau, Germany; 5 Klinikum Chemnitz gGmbH, Chemnitz, Germany; 6 Aichi Cancer Center Hospital, Aichi, Japan; 7 St George’s University, London, UK; 8 University of Melbourne and St. Vincent’s Hospital, Melbourne, Australia; 9 Bristol Haematology and Oncology Centre, Bristol, UK; 10 James Paget Hospital, Great Yarmouth, UK; 11 ICO-IJC-Hospital Germans Trias i Pujol, Barcelona, Spain; 12 Medical and Health Science Centre, University of Debrecen, Debrecen, Hungary; 13 Uniklinik Heidelberg, Heidelberg, Germany; 14 F. Hoffmann-La Roche Ltd, Basel, Switzerland

**Keywords:** Follicular lymphoma, Indolent non-Hodgkin lymphoma, Obinutuzumab, Quality of life, Rituximab

## Abstract

Health-related quality of life was a secondary endpoint in the phase III GALLIUM study in previously untreated patients with follicular lymphoma who were treated with rituximab- or obinutuzumab-chemotherapy. Patients were randomized 1:1 to receive induction therapy with obinutuzumab- or rituximab-chemotherapy and maintenance in responders. Health-related quality of life was assessed using the Functional Assessment of Cancer Treatment-Lymphoma questionnaire, incorporating well-being and lymphoma-specific subscales. Assessments were performed at baseline, and during induction, maintenance, and follow-up (maximum 84 months). Clinically meaningful responses were defined by minimally important difference values. Of 1202 randomized patients (median follow-up 57.4 months), 557/601 (92.7%; obinutuzumab-chemotherapy) and 548/601 (91.2%; rituximab-chemotherapy) completed all Functional Assessment of Cancer Treatment-Lymphoma scales at baseline. Mean baseline health-related quality of life scores were similar between both arms, with all patients having some functional impairment and lymphoma symptoms. Over the course of treatment, mean health-related quality of life remained similar in both arms. Equal proportions of patients in both arms achieved minimally important difference by the Functional Assessment of Cancer Treatment-Lymphoma lymphoma-specific subscale and summary scales throughout induction, maintenance, and follow-up. On each summary scale, ~ 50% of patients in each arm achieved minimally important difference by maintenance month 2. In GALLIUM, similar improvements in health-related quality of life were seen with obinutuzumab- and rituximab-chemotherapy, suggesting that both treatments reduced lymphoma-related symptoms, and treatment-related side effects did not abrogate these improvements in well-being. ClinicalTrials.gov identifier: NCT01332968.

**Electronic supplementary material:**

The online version of this article (10.1007/s00277-020-04021-6) contains supplementary material, which is available to authorized users.

## Introduction

Follicular lymphoma (FL) encompasses approximately 70% of indolent non-Hodgkin lymphomas (iNHL) and 22% of all NHLs [1]. Rituximab (R), a type I monoclonal antibody (mAb), plus chemotherapy (R-chemo) induction, and maintenance therapy has resulted in improved outcomes for patients with FL [2–5]. Nevertheless, 20–35% of patients experience progressive disease, relapse, or death within 2 years [4, 6], with early disease progression associated with worse prognosis [7, 8].

Obinutuzumab (GA101; G) is the first glycoengineered, type II, humanized anti-CD20 mAb, promoting enhanced antibody-dependent cellular toxicity and direct cell death against B cell malignancies when compared with type I mAbs [9]. The phase III GALLIUM study (NCT01332968) evaluated G-chemotherapy (G-chemo) versus R-chemo as induction therapy in patients with previously untreated, advanced-stage FL, followed by maintenance with the same antibody in responders. In the primary analysis of this study (data cut-off January 31, 2016), the primary endpoint of investigator-assessed progression-free survival (PFS) was met, with an improvement in PFS shown for patients treated with G-chemo versus those treated with R-chemo (median follow-up, 34.5 months; hazard ratio 0.66; 95% confidence interval 0.51–0.85; *p* = 0.001) [10]. Adverse events (AEs) were consistent with the known safety profiles of both study treatments [10].

Despite the improvements in patient outcomes that have come with advancements in immunochemotherapy, the treatment of conditions like FL can often have a more negative impact on the patient than the disease itself [11]. Meaningful improvements in health-related quality of life (HRQoL) and the effect that serious or persistent treatment-related symptoms have on patients are important factors to consider, notably with the increased chances of prolonged survival following treatment.

Several studies have investigated the impact of treatment on HRQoL in patients with iNHL. In a population of patients with FL, those who were newly diagnosed with active disease had similar HRQoL compared with patients that were either in partial/complete remission or disease free, while having better HRQoL than those who had relapsed [11]. In the phase III GADOLIN trial of patients with relapsed/refractory iNHL, patients treated with G-bendamustine (B) versus B had a delayed time to worsening and more reports of clinically meaningful improvements in HRQoL [12].

However, previous analyses have primarily been cross-sectional studies, with none having compared G-chemo with R-chemo in a population of patients with FL. In this secondary analysis of the GALLIUM study, we compared the changes in HRQoL in first-line patients with FL treated with G-chemo or R-chemo, investigating the potential differences in patient-reported outcomes (PROs) between the two treatments.

## Methods

### Study design

GALLIUM was a phase III, open-label, parallel-group study. Patients with previously untreated grade 1–3a FL were randomized 1:1 to receive induction therapy with G plus chemotherapy (cyclophosphamide, doxorubicin, vincristine, and prednisone [CHOP]; cyclophosphamide, vincristine, and prednisone [CVP]; or B). Six to eight cycles of chemotherapy were prescribed, depending on the selected chemotherapy (chosen upfront by investigators at each site; all patients at the same site received the same regimen). Patients achieving at least a partial response on contrast-enhanced computed tomography received maintenance with the same antibody for 2 years or until progressive disease.

Patient selection, study methods, and treatment are described in detail within the Online Resource (Methods) and elsewhere [10].

### Patient-reported HRQoL assessments

HRQoL was assessed using the Functional Assessment of Cancer Treatment-Lymphoma (FACT-Lym) questionnaire [13], incorporating the FACT-General (FACT-G) scale (physical well-being [PWB], functional well-being [FWB], emotional well-being [EWB], and social/family well-being [SWB]) and the FACT-Lym lymphoma-specific (LYMS) subscale. Summary scales FACT-Lym trial outcome index (TOI), FACT-G, and Total (TOT) were also calculated. Further details of these questionnaires can be found within the Online Resource (Methods).

Assessments were performed at baseline (cycle [C]1 day [D]1), C3D1, end of induction, during maintenance months 2 and 12, at the end of maintenance (month 24), and then every 12 months during follow-up, up to 84 months.

Clinically meaningful responses were defined by minimally important difference (MID) values in FACT-Lym and FACT-G. A MID reflects the smallest difference in a score that is considered to be clinically important to the patient [14–16]. Patients who achieved an improved score versus baseline, reaching the upper limits of FACT-Lym (LYMS, ≥ 3 points; TOI, ≥ 6 points; TOT, ≥ 7 points) and FACT-G (≥ 2–3, excluding the SWB scale), were classified as responders, with differences between treatment groups assessed [15, 16]. Higher FACT-Lym scores indicate improved functioning, HRQoL, and health status.

### Statistical analysis

PRO analyses included all randomized patients who had a baseline measure and ≥ 1 post-baseline assessment. Missing values were not imputed; however, prorated scores were calculated based on developed guidelines [17]. Analyses were performed separately for patients with FL versus the overall population (data cut-off February 12, 2018).

Questionnaire completion rates were calculated. For each FACT-Lym questionnaire scale, descriptive statistics at each visit and changes from baseline are presented. After baseline FACT-Lym LYMS, TOI, TOT, and FACT-G were evaluated, mean score changes from baseline were calculated for each time point. Finally, the proportion of patients with FL achieving the MID for each scale, i.e., a clinically meaningful response, was assessed.

Here we present only the results up to month 48, due to the dense censoring that occurs after this time point; results up to month 84 can be found within the Online Resource.

## Results

### Disposition and updated analysis

In total, 1202 patients with FL were randomized to receive either G-chemo (*n* = 601) or R-chemo (*n* = 601) in the GALLIUM trial (Online Resource: Supplementary Fig. 1). Baseline demographic and disease characteristics were well-balanced between arms [10].

The efficacy and safety findings of the updated GALLIUM analysis (data cut-off February 12, 2018; median follow-up, 57.4 months) were consistent with the primary analysis; G-chemo continued to provide clinically meaningful improvements in PFS relative to R-chemo (4-year PFS rate, 78.1% vs. 67.2%; hazard ratio 0.73; 95% confidence interval 0.59–0.90; *p* = 0.0034) [18]. No new safety signals were identified, with grade 3–5 AEs and serious AEs being more common in patients treated with G-chemo versus R-chemo, in line with the primary analysis [10, 18].

### Questionnaire completion rates

**Fig. 1:**
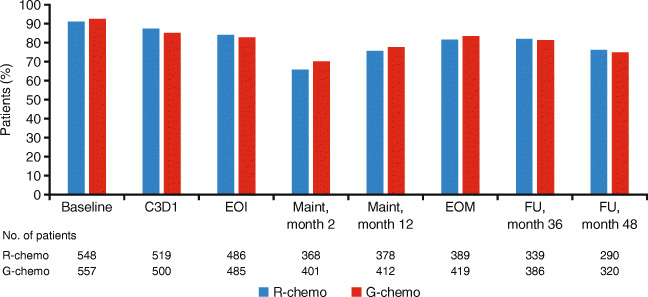
FACT-Lym questionnaire completion data for R-chemo vs. G-chemo. The table below the graph shows the number of patients still receiving treatment who completed all FACT-Lym scales at the specified time point. *C*, cycle; *chemo*, chemotherapy; *D*, day; *EOI*, end of induction; *EOM*, end of maintenance; *FACT-Lym*, Functional Assessment of Cancer Treatment-Lymphoma; *FU*, follow-up; *G*, obinutuzumab; *Maint*, maintenance; *R*, rituximab

In the G-chemo and R-chemo arms, 557/601 (92.7%) and 548/601 (91.2%) patients completed all scales of FACT-Lym assessments at baseline, respectively. Throughout the study, the percentage of patients who completed all scales of FACT-Lym was well-balanced between arms. Although completion rates did decline over the course of treatment, low rates of attrition during induction, maintenance, and follow-up (up to month 48) were seen (Fig. 1). The proportion of patients completing FACT-Lym assessments decreased beyond this time point, and patient numbers declined substantially up to follow-up month 84 (Online Resource: Supplementary Table 1).

### Absolute and mean change in HRQoL questionnaire scores from baseline

Mean baseline values for FACT-Lym composite scores TOI and TOT and FACT-Lym LYMS subscale and individual FACT-G subscales (PWB, FWB, EWB, and SWB) were comparable across treatment arms (Online Resource: Supplementary Fig. 2). In both treatment arms, patients exhibited some level of baseline impairment according to functioning and lymphoma symptom subscales, noted by mean scores between 5 and 15 points below the possible maximum (depending on the subscale).

**Fig. 2:**
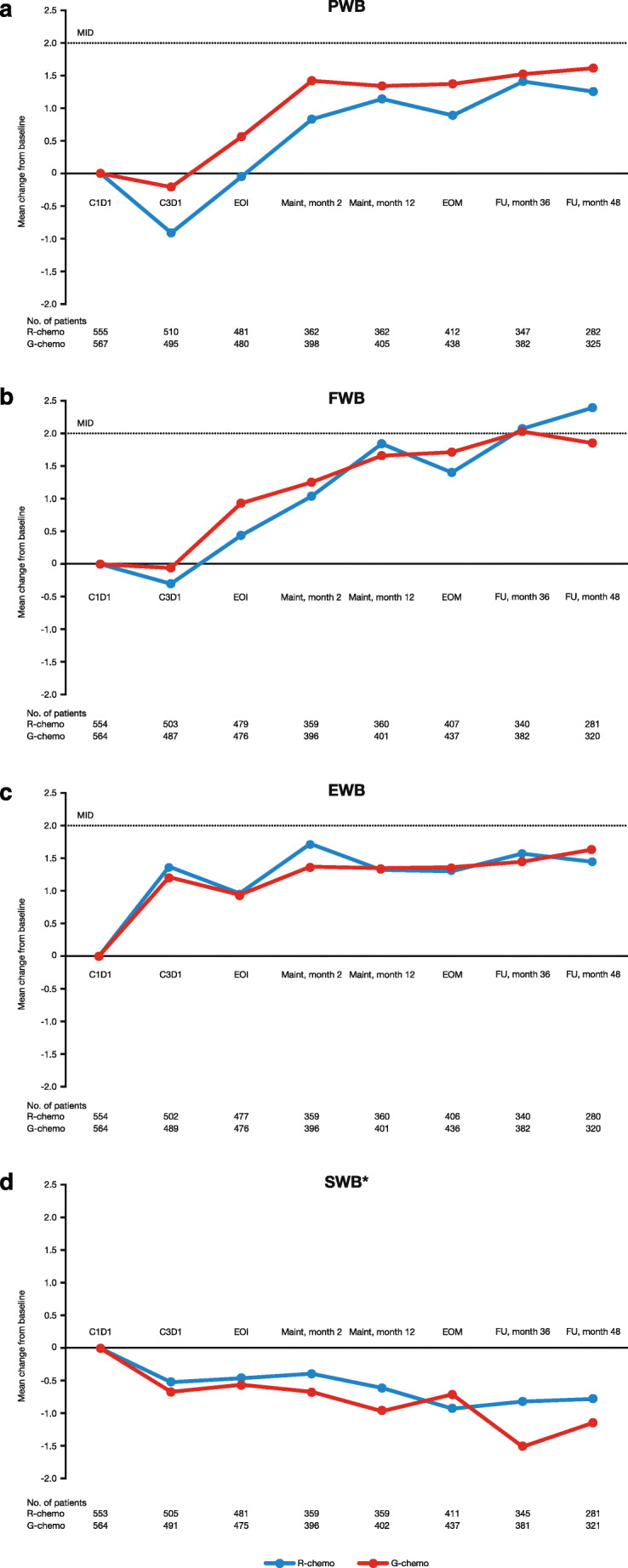
Mean change from baseline in FACT-G PWB, FWB, EWB, and SWB, by treatment arm. **a** PWB, **b** FWB, **c** EWB, and **d** SWB. *Changes of 2–3 points are considered meaningful on the PWB, FWB, and EWB scales. A meaningful change has not yet been defined for the SWB scale. The number of patients still receiving treatment who completed the FACT-G questionnaire at the specified time point is specified below the graph. *C*, cycle; *chemo*, chemotherapy; *D*, day; *EOI*, end of induction; *EOM*, end of maintenance; *EWB*, emotional well-being; *FACT-G*, Functional Assessment of Cancer Treatment-General; *FU*, follow-up; *FWB*, functional well-being; *G*, obinutuzumab; *Maint*, maintenance; *MID*, minimally important difference; *PWB*, physical well-being; *R*, rituximab; *SWB*, social/family well-being

For FACT-G assessments, an initial negative mean change from baseline (C1D1) by PWB and FWB subscales was reported (C3D1, G-chemo vs. R-chemo, PWB – 0.21 vs. – 0.91; FWB – 0.06 vs. – 0.30; Fig. 2a and b, respectively). Conversely, EWB increased rapidly by the first time point in both treatment arms (C3D1, G-chemo vs. R-chemo, 1.36 vs. 1.49; Fig. 2c). Modest increases were then reported in both treatment arms for the PWB, FWB, and EWB scales up to follow-up month 48. SWB scores decreased versus baseline for both G-chemo and R-chemo (follow-up month 48, − 1.15 vs. – 0.78; Fig. 2d). Despite the increases seen for PWB, FWB, and EWB, scores only exceeded the threshold for clinically meaningful change on the FWB subscale (> 2) for patients treated with R-chemo. Beyond follow-up month 48, PWB scores continued to increase, with MID (> 2–3) achieved between months 72 and 84 (Online Resource: Supplementary Table 2).

**Fig. 3:**
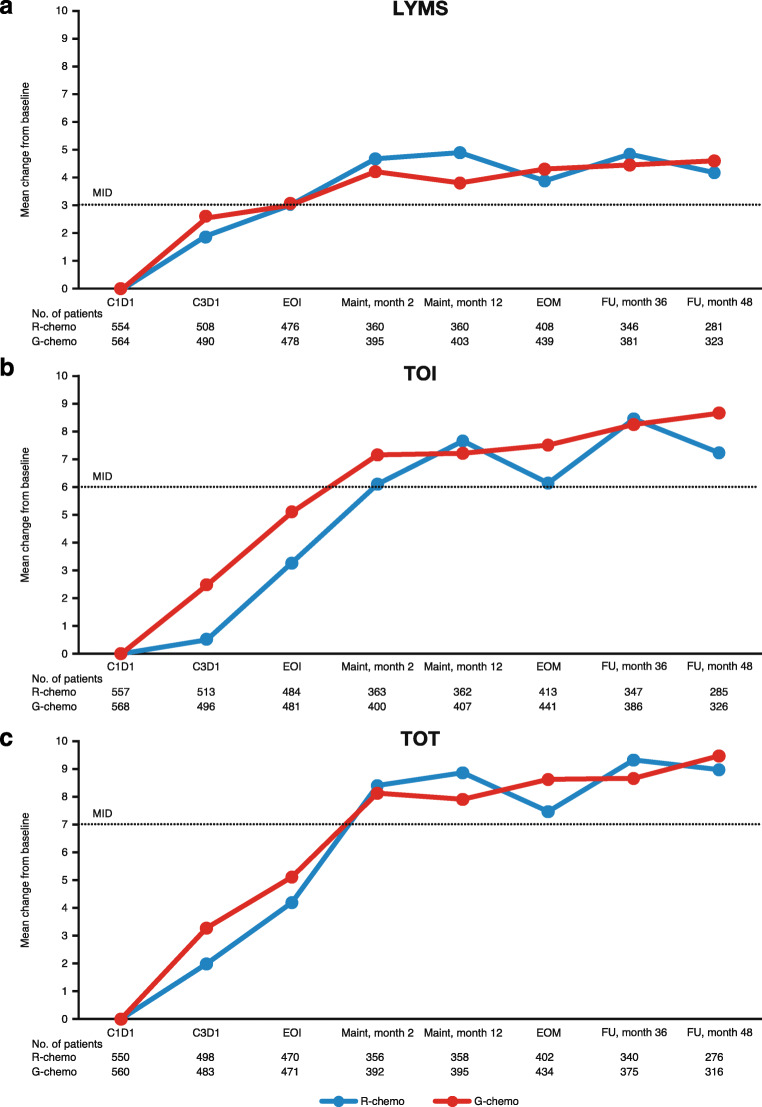
Mean change from baseline in FACT-Lym LYMS, TOI, and TOT scores, by treatment arm. **a** LYMS, **b** TOI, and **c** TOT. The number of patients still receiving treatment who completed the FACT-Lym questionnaire at the specified time point is specified below the graph. *C*, cycle; *chemo*, chemotherapy; *D*, day; *EOI*, end of induction; *EOM*, end of maintenance; *FACT-Lym*, Functional Assessment of Cancer Treatment-Lymphoma; *FU*, follow-up; *G*, obinutuzumab; *LYMS*, lymphoma-specific; *Maint*, maintenance; *MID*, minimally important difference; *R*, rituximab; *TOI*, trial outcome index; *TOT*, total

Over the course of treatment, similar trends were observed in patients treated with G-chemo and R-chemo up to follow-up month 48. A rapid increase in FACT-Lym LYMS was seen from baseline to the first time point for both G-chemo and R-chemo (C3D1, 2.73 vs. 2.04, respectively). The mean change from baseline continued to increase at each time point for FACT-Lym LYMS (Fig. 3a), TOI (Fig. 3b), and TOT (Fig. 3c), up to maintenance month 2, at which point MID was achieved (G-chemo vs. R-chemo, LYMS [≥ 3] 4.52 vs. 4.80; TOI [≥ 6] 7.17 vs. 6.22; and TOT [≥ 7] 8.13 vs. 8.40, respectively). From maintenance month 2 up to follow-up month 48, the mean change from baseline scores levelled off for all scales (for G-chemo vs. R-chemo, LYMS 4.76 vs. 4.50; TOI 8.51 vs. 7.23; TOT 9.48 vs. 8.98, respectively). Mean changes from baseline continued to increase up to follow-up month 84 in all scales in both arms, excluding the FACT-Lym LYMS assessment in patients treated with R-chemo (Online Resource: Supplementary Table 3).

### Clinically meaningful improvement in FACT-Lym LYMS subscale and FACT-Lym composite (TOI and TOT) scores

**Fig. 4:**
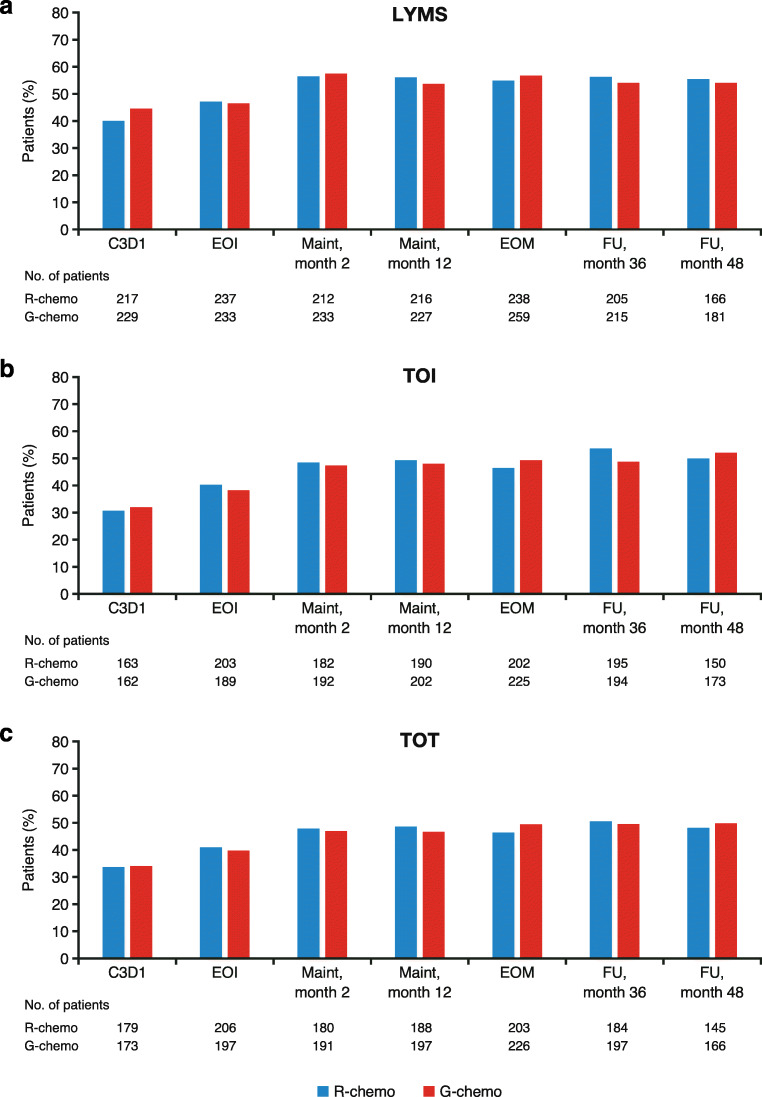
Proportion of patients with FL achieving MID on FACT-Lym LYMS score (≥ 3), TOI score (≥ 6), and TOT score (≥ 7). **a** LYMS (≥ 3), **b** TOI (≥ 6), and **c** TOT (≥ 7) score. The number of patients still receiving treatment who achieved MID at the specified time point is specified below the graph. *C*, cycle; *chemo*, chemotherapy; *D*, day; *EOI*, end of induction; *EOM*, end of maintenance; *FACT-Lym*, Functional Assessment of Cancer Treatment-Lymphoma; *FL*, follicular lymphoma; *FU*, follow-up; *G*, obinutuzumab; *LYMS*, lymphoma-specific; *Maint*, maintenance; *MID*, minimally important difference; *R*, rituximab; *TOI*, trial outcome index; *TOT*, total

Equal proportions of patients in the G-chemo and R-chemo arms achieved MID by the FACT-Lym LYMS score, and the composite scores TOI and TOT, throughout induction, maintenance, and follow-up (Fig. 4a, b, and c, respectively). At the first assessment (C3D1), ≥ 30% of patients evaluated achieved MID, with approximately 50% of patients reporting clinically meaningful improvements at maintenance month 2 and continuing to respond at follow-up month 48 (G-chemo vs. R-chemo, LYMS, 54.2% vs. 55.5%; TOI, 51.8% vs. 49.7%; TOT, 49.7% vs. 48.0%, respectively). Results beyond follow-up month 48 are reported in Online Resource: Supplementary Table 4*.*

## Discussion

For first-line patients with FL, treatment with G-chemo results in improved PFS when compared with the current standard-of-care treatment, R-chemo [10, 18]. However, achieving improvements in PROs is just as important as improving clinical outcomes such as PFS. In the current analysis of the GALLIUM study, similar improvements in HRQoL were seen with G-chemo and R-chemo treatment in patients with FL, with no clear differences between treatment groups reported at any time point. Within the context of improved PFS, these results further support the positive benefit-risk balance of G-chemo over R-chemo in previously untreated patients with FL.

In the current study, PWB, FWB, EWB, and SWB scores were similar at baseline, albeit lower than previously reported values in patients with newly diagnosed active FL. In a study by Pettengell et al. of patients with newly diagnosed active disease, scores were higher in all FACT-Lym subscales (range 0–8 points) versus those enrolled in GALLIUM [11]. It is important to note that some of the active disease-newly diagnosed group were on a watch and wait strategy, which could indicate a lower disease burden compared with the patients enrolled in GALLIUM [11]. A study of HRQoL in long-term survivors of iNHL and aggressive NHL found that, at diagnosis, patients with stage III/IV aggressive NHL had significantly worse HRQoL than those with stage I/II aggressive or iNHL [19]. These findings demonstrate that first-line patients with FL may experience an improved HRQoL compared with patients with relapsed disease (although worsened compared with the general population [20]) and support the view that HRQoL differs according to disease state.

Over the course of treatment, similar improvements in FACT-Lym composite scores, and thus HRQoL, were observed in both treatment arms. At no time point up to follow-up, month 48 was the average HRQoL of patients receiving G-chemo clinically worse than those receiving R-chemo. Patients in both arms experienced clinically meaningful improvements in FACT-Lym LYMS and in the summary scales (i.e., TOI and TOT). These results suggest that lymphoma-related symptoms improved in both treatment arms to a degree recognizable by patients, subsequently driving improvement in composite summary scales. Importantly, this was despite the higher AE rates observed in the G-chemo arm as reported in both the primary and updated analyses of GALLIUM [10, 18].

Findings are further supported by the number of patients reporting clinically meaningful improvements in both treatment arms, with approximately half having achieved a MID by maintenance month 2, sustained up to follow-up month 48. When coupled with lack of deterioration in PWB and FWB, these results suggest that improvements in well-being were not abrogated by the increased number of treatment-related side effects reported in patients receiving G-chemo versus R-chemo. This is in line with results reported from the GADOLIN study of patients with relapsed/refractory iNHL, whereby patients treated with G-B had improved HRQoL scores when compared with those treated with B alone, and benefits in PFS seen with G-B were not abrogated by treatment-related toxicity [12]. Previously, the majority of patients who survived iNHL still feared the probability of relapse and second malignancy [19]. Therefore, patients who have responded to therapy should still be monitored post-response to ensure improvements in HRQoL are sustained. In the current analysis, slight improvements in average scores, though less than the MID, were seen with PWB, FWB, and EWB scores, with very small deterioration (approximately − 1 point) seen in the SWB scores, throughout the study period in both treatment arms. When interpreting these data, it is important to consider that GALLIUM was an open-label study, and patients were aware of their treatment regimen. It is possible that the initial post-treatment HRQoL scores may reflect a certain degree of expectation toward treatment outcome, which may have impacted patients’ reporting of HRQoL in either treatment arm. Any influence that knowledge of therapy may have had on patient reporting of HRQoL was likely to have resolved over the course of treatment as patients either experienced or did not experience improvements in their condition.

At the first time point in the GALLIUM and GADOLIN trials (C3D1 and C5D1, respectively), a decline compared with baseline in both PWB and FWB was reported, with modest increases reported thereafter [10, 12]. As patients enrolled in GALLIUM were previously untreated, this decline indicates further measures should be taken during early induction to ensure the provision of G plus combination therapy does not negatively influence physical and functional well-being.

In GALLIUM, the chemotherapy regimen was selected upfront by each participating center, with all patients at the same center receiving the same regimen (i.e., B, CHOP, or CVP). The results reported here do not address differences in HRQoL that may have been seen between chemotherapy regimens. Furthermore, patients received either six or eight cycles of chemotherapy depending on chemotherapy regimen, which may also have led to subgroup differences in HRQoL that have not been addressed here. Additionally, there is the possibility that the length of time between assessments may have missed changes that occurred in symptom burden during the initial weeks of treatment; a finding that has been noted outside of hematology studies (i.e., non-small-cell lung cancer during the first week following chemotherapy treatment) [21]. Therefore, the timing of HRQoL assessment should be carefully considered in the design of future trials, as this may influence the chances of detecting differences between treatment regimens.

It should be noted that questionnaire completion rates were high at baseline, and a low rate of attrition was observed throughout the study in both treatment arms. This suggests that issues with non-compliance should not deter the implementation of PRO measures in the design of future clinical trials. In addition, the high compliance rates provide confidence in the representativeness of the HRQoL in patients treated with G-chemo and R-chemo in the GALLIUM trial.

This analysis of the secondary endpoint HRQoL of the GALLIUM study sought to compare changes in HRQoL in first-line patients with FL treated with G- or R-based chemotherapy. These results demonstrate that aspects of HRQoL and lymphoma symptoms improved over the course of treatment, and there were a high number of patients in both treatment arms that experienced a clinically meaningful improvement in HRQoL. In addition, as PRO scores were similar between arms throughout induction, maintenance, and follow-up, treatment-related toxicity did not abrogate improvements in well-being in those patients who did not experience clinically meaningful responses. Furthermore, there was no evidence of cumulative side effects. Both clinical outcomes and PROs should be considered in future clinical trials in patients with FL.

## Electronic supplementary material

ESM 1 (PDF 323 kb)

## Data Availability

Qualified researchers may request access to individual patient level data through the clinical study data request platform (www.clinicalstudydatarequest.com). Further details on Roche’s criteria for eligible studies are available here (https://clinicalstudydatarequest.com/Study-Sponsors/Study-Sponsors-Roche.aspx). For further details on Roche’s Global Policy on the sharing of clinical information and how to request access to related clinical study documents, see here (https://www.roche.com/research_and_development/who_we_are_how_we_work/clinical_trials/our_commitment_to_data_sharing.htm).
