# Supplementary material for: Health-related quality of life in the phase III GALLIUM study of obinutuzumab- or rituximab-based chemotherapy in patients with previously untreated advanced follicular lymphoma
Source: Ann Hematol. 2020 Apr 20;99(12):2837–46. doi: 10.1007/s00277-020-04021-6 (PMC7683459; doi:10.1007/s00277-020-04021-6)
Supplement: Supplementary file 1 — (PDF 323 kb) [file 277_2020_4021_MOESM1_ESM.pdf]

SUPPLEMENTARY MATERIAL

Article title: Health-related quality of life in the phase III GALLIUM study of obinutuzumab- or rituximab-based chemotherapy in patients with previously untreated advanced follicular lymphoma

Journal name: Annals of Hematology

Author names: Andrew Davies, Peter Trask, Judit Demeter, Axel Florschütz, Mathias Hänel, Tomohiro Kinoshita, Ruth Pettengell, Hang Quach, Stephen Robinson, Shalal Sadullah, Juan-Manuel Sancho, Miklos Udvardy, Mathias Witzens-Harig, Andrea Knapp, and Wenxin Liu

Corresponding author: Andrew Davies, Cancer Research UK Centre, University of Southampton, a.davies@southampton.ac.uk

SUPPLEMENTARY MATERIAL CAPTIONS

Methods

Study design

Patient-reported health-related quality of life (HRQoL) assessments

References

Supplementary tables

Supplementary Table 1 Questionnaire completion rates beyond follow-up month 48

Supplementary Table 2 Mean change from baseline in FACT-G PWB, FWB, EWB, and SWB beyond FU month 48

Supplementary Table 3 Mean change from baseline in FACT-Lym LYMS, TOI, and TOT scores beyond FU month 48

Supplementary Table 4 Proportion of patients achieving clinically meaningful responses by LYMS, TOI, and TOT beyond FU month 48

Supplementary figure

Supplementary Fig. 1 Patient disposition in all patients with FL in GALLIUM

Supplementary Fig. 2 Baseline FACT-Lym scores for FACT-G and FACT-Lym LYMS, TOI, and TOT. (a) FACT-G and (b) FACT-Lym LYMS, TOI, and TOT

Methods

Study design

GALLIUM was a phase III, open-label, randomized, parallel-group study. Patients with previously untreated grade 1–3a follicular lymphoma (FL) were randomized 1:1 to receive induction therapy with obinutuzumab (G; 1000 mg on days [D]1, 8, and 15 of cycle [C]1 and D1 of subsequent cycles) or rituximab (R; 375 mg/m^2 on D1 of each C) plus chemotherapy (cyclophosphamide, doxorubicin, vincristine, and prednisone [CHOP], cyclophosphamide, vincristine, and prednisone [CVP], or bendamustine).

Eligible patients were aged ≥ 18 years with histologically documented, previously untreated, CD20-positive, advanced-stage (stage III/IV or stage II with bulky disease [≥ 7 cm]) FL, with an Eastern Cooperative Oncology Group (ECOG) performance status of 0–2, requiring treatment as per Group d'Etude des Lymphomes Folliculaires criteria.

Patient-reported health-related quality of life (HRQoL) assessments

The Functional Assessment of Cancer Treatment-Lymphoma (FACT-Lym) questionnaire was developed and psychometrically validated to assess several aspects of HRQoL in patients with lymphoma.[1,2,3] FACT-Lym, comprising the 27-item FACT-General (FACT-G) scale and the 15-item FACT-Lym lymphoma-specific (LYMS) subscale, is scored on a 5-point scale (0 = "not at all" to 4 = "very much"). FACT-G assesses four aspects of HRQoL: physical well-being (PWB, 7 items), functional well-being (FWB, 7 items), emotional well-being (EWB, 6 items), and social/family well-being (SWB, 7 items). The FACT-Lym LYMS subscale includes

questions related to symptoms (e.g., pain, swelling, fever, etc.) and additional patient concerns.[4]

References

1. Webster K, Cashy J, Cella D. Measuring quality of life (QOL) in patients with non-Hodgkin's lymphoma (NHL): the Functional Assessment of Cancer Therapy-LYmphoma (FACT-LYM). Qual Life Res. 2005;14(9):2103.

2. Hlubocky FJ, Webster K, Cashy J, Beaumont J, Cella D. The development and validation of a measure of health-related quality of life for non-hodgkin's lymphoma: The Functional Assessment of Cancer Therapy—Lymphoma (FACT-Lym). Lymphoma. 2013;2013:1–9.

3. Cella D, Webster K, Cashy J, Kutikova L, Burgess MF, Lin BK, et al. Development of a measure of health-related quality of life for non-hodgkin’s lymphoma clinical research: The Functional Assessment of Cancer Therapy - Lymphoma (FACT-Lym). Blood. 2005;106:750.

4. Harrison CN, Mesa RA, Kiladjian JJ, Al-Ali HK, Gisslinger H, Knoops L, et al. Health-related quality of life and symptoms in patients with myelofibrosis treated with ruxolitinib versus best available therapy. Br J Haematol. 2013;162(2):229–239.

Supplementary Table 1 Questionnaire completion rates beyond follow-up month 48

Completion rate, *n* (%)	R-chemo	G-chemo
FU, month 60	126 (67.7)	132 (68.4)
FU, month 72	28 (52.8)	30 (53.6)
FU, month 84	4 (57.1)	6 (100)

FU, follow-up; *G-chemo*, obinutuzumab plus chemotherapy; *R-chemo*, rituximab plus chemotherapy

Supplementary Table 2 Mean change from baseline in FACT-G PWB, FWB, EWB, and SWB beyond FU month 48

	R-chemo		G-chemo	
	n	Mean change from baseline	*n*	Mean change from baseline
PWB				
FU, month 60	130	1.28	140	1.41
FU, month 72	30	2.13	33	1.85
FU, month 84	6	3.42	6	3.17
FWB				
FU, month 60	127	1.75	141	2.73
FU, month 72	30	2.04	32	1.01
FU, month 84	6	3.83	6	1.83
EWB				
FU, month 60	128	2.36	141	2.02
FU, month 72	30	1.42	32	1.59
FU, month 84	6	2.10	6	1.50
SWB				
FU, month 60	129	–1.37	140	–0.84
FU, month 72	30	–0.27	33	–1.96
FU, month 84	5	–0.77	6	–0.61

EWB, emotional well-being; *FACT-G*, Functional Assessment of Cancer Treatment-General; *FU*, follow-up; *FWB*, functional well-being; *G-chemo*, obinutuzumab plus chemotherapy; *PWB*, physical well-being; *R-chemo*, rituximab plus chemotherapy; *SWB*, social/family well-being

Supplementary Table 3 Mean change from baseline in FACT-Lym LYMS, TOI, and TOT scores beyond FU month 48

	R-chemo		G-chemo	
	n	Mean change from baseline	*n*	Mean change from baseline
LYMS				
FU, month 60	129	4.72	139	5.01
FU, month 72	30	5.33	32	5.76
FU, month 84	6	3.92	6	6.17
TOI				
FU, month 60	131	7.85	143	9.45
FU, month 72	30	9.50	33	9.45
FU, month 84	6	11.17	6	11.17
TOT				
FU, month 60	126	9.47	136	10.29
FU, month 72	30	10.81	31	7.88
FU, month 84	6	13.92	6	12.39

FACT-Lym, Functional Assessment of Cancer Treatment-Lymphoma; *FU*, follow-up; *G-chemo*, obinutuzumab plus chemotherapy; *LYMS*, lymphoma-specific; *R-chemo*, rituximab plus chemotherapy; *TOI*, trial outcome index; *TOT*, total

Supplementary Table 4 Proportion of patients achieving clinically meaningful responses by LYMS, TOI, and TOT beyond FU month 48

Patients achieving MID, *n* (%)	R-chemo	G-chemo
LYMS (> 3-point improvement)		
FU, month 60	81 (59.1)	81 (54.7)
FU, month 72	22 (73.3)	21 (60.0)
FU, month 84	4 (66.7)	2 (33.3)
TOI (> 6-point improvement)		
FU, month 60	72 (52.2)	83 (55.7)
FU, month 72	15 (50.0)	14 (40.0)
FU, month 84	3 (50.0)	2 (33.3)
TOT (> 7-point improvement)		
FU, month 60	70 (50.7)	74 (49.7)
FU, month 72	18 (60.0)	13 (37.1)
FU, month 84	4 (66.7)	2 (33.3)

FU, follow-up; *G-chemo*, obinutuzumab plus chemotherapy; *LYMS*, lymphoma-specific; *MID*, minimally important difference; *R-chemo*, rituximab plus chemotherapy; *TOI*, trial outcome index; *TOT*, total

Supplementary Fig. 1 Patient disposition in all patients with FL in GALLIUM

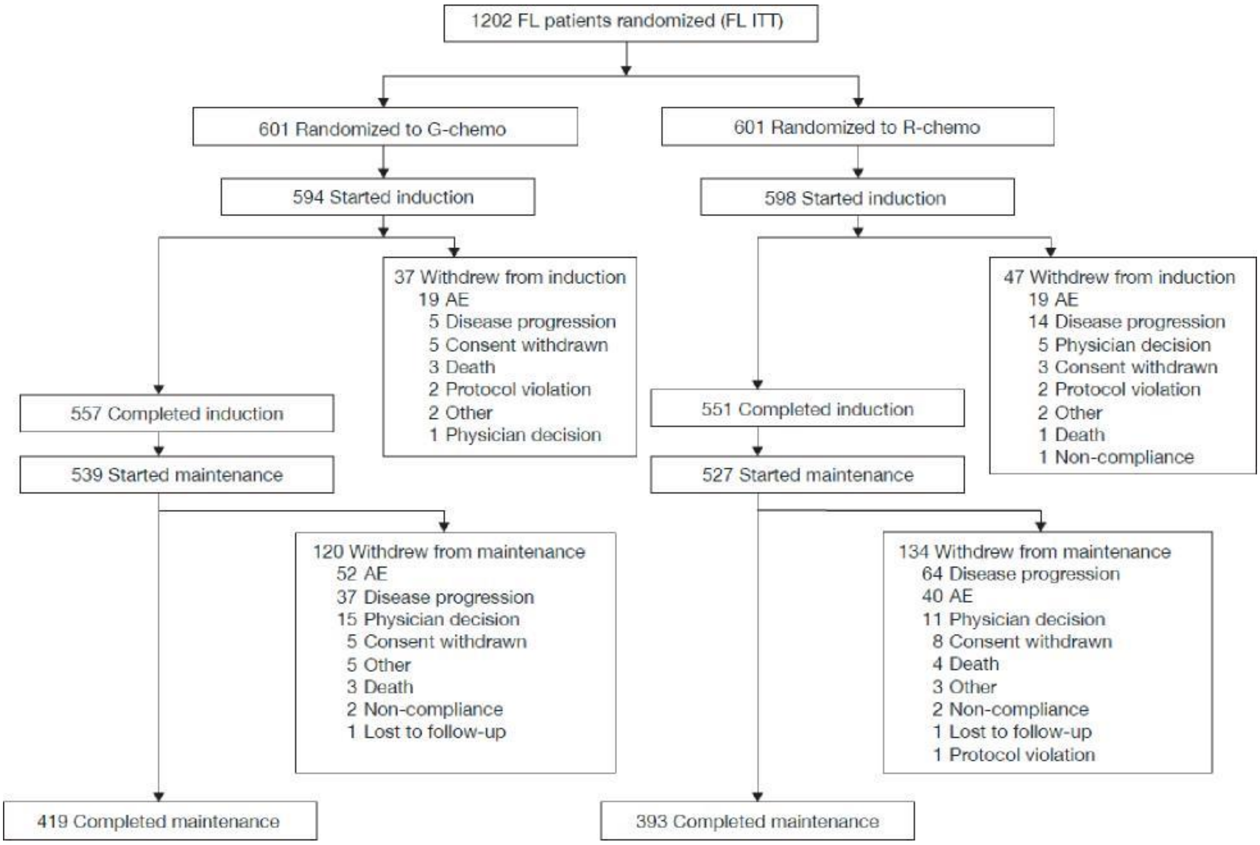

AE, adverse event; *FL*, follicular lymphoma; *G-chemo*, obinutuzumab plus chemotherapy; *ITT*, intent-to-treat; *R-chemo*, rituximab plus chemotherapy

Supplementary Fig. 2 Baseline FACT-Lym scores for FACT-G and FACT-Lym LYMS, TOI, and TOT. (a) FACT-G and (b) FACT-Lym LYMS, TOI, and TOT

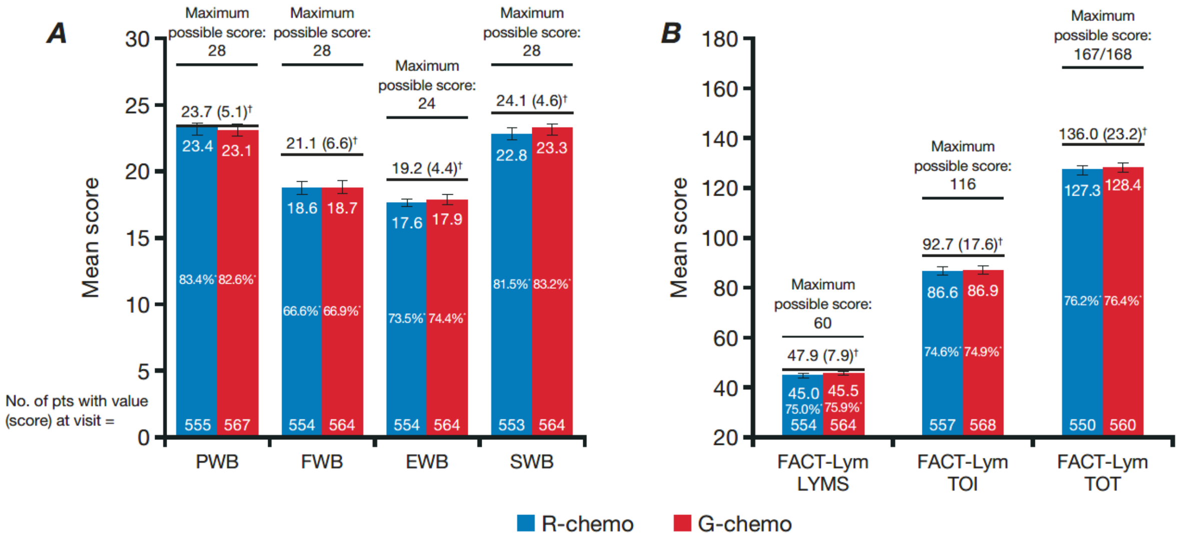

*Denote the percentage value of the total range for each scale. Higher scores denote improved HRQoL or well-being vs. lower scores; †reference score (SD) in the newly diagnosed FL population, adapted from Pettengell et al. (2008).[11]

Chemo, chemotherapy; *EWB*, emotional well-being; *FACT-G*, Functional Assessment of Cancer Treatment-General; *FACT-Lym*, FACT-Lymphoma; *FL*, follicular lymphoma; *FWB*, functional well-being; *G*, obinutuzumab; *HRQoL*, health-related quality of life; *LYMS*, lymphoma-specific; *pts*, patients; *PWB*, physical well-being; *R*, rituximab; *SD*, standard deviation; *SWB*, social/family well-being; *TOI*, trial outcome index; *TOT*, total
